# Spontaneous Intramural Duodenal Hematoma: A Rare Complication of Pancreatitis

**DOI:** 10.7759/cureus.8491

**Published:** 2020-06-07

**Authors:** Ravi Kumar, Pal Satyajit Singh Athwal, Mukesh Kumar, Kanchan Devi, Sukhmanii Kahlon

**Affiliations:** 1 Internal Medicine, Jinnah Sindh Medical University, Karachi, PAK; 2 Internal Medicine, Saraswathi Institute of Medical Sciences, Hapur, IND; 3 Neurology, Shaheed Mohtarma Benazir Bhutto Medical University, Larkana, PAK; 4 Internal Medicine, Medical University of the Americas, Camps, KNA

**Keywords:** intramural duodenal hematoma, pancreatic origin, pancreatitis

## Abstract

Intramural duodenal hematoma is an uncommon entity, usually associated with trauma. Spontaneous intramural duodenal hematoma is an even more rare phenomenon reported in patients with anticoagulation therapy, gastrointestinal endoscopy procedure or coagulopathy. We report a case of spontaneous intramural duodenal hematoma in a 30-year-old male as a pancreatitis complication, very few cases have been known in the past and still a lot is to be discovered about this rare hematoma associated with pancreatitis. This condition can have catastrophic consequences and should be managed appropriately.

## Introduction

In 1838, during an autopsy, McLauchlan first introduced intramural duodenal hematoma [1]. Hematoma is formed inside the wall of duodenum and is usually seen in trauma patients. Intramural duodenal hematoma can lead to gastrointestinal obstruction, perforation, intussusception which can be life threatening. Spontaneous intramural duodenal hematoma cases are very scarcely reported in the past which were linked to coagulation disorders, anticoagulation therapy and endoscopy procedures [2,3]. Spontaneous intramural duodenal hematoma secondary to pancreatic pathology is associated with pancreatic carcinoma, chronic pancreatitis and ectopic pancreas [4]. Pathophysiology and prognosis of spontaneous intramural hematoma of pancreatic origin remain unknown. We report a case of spontaneous intramural duodenal hematoma in 30-year-old male patient with history of pancreatitis three days ago.

## Case presentation

A 30-year-old-male with history of alcohol abuse presented with a complaint of severe epigastric pain. The pain was diffuse across his abdomen, 10/10 in intensity and sharp in quality. The patient was diagnosed with acute pancreatitis and was discharged three days ago. On physical examination, everything was normal except diffuse abdominal tenderness. A nasogastric tube was inserted with improvement in his pain along with fluid management. Analgesics in the form of morphine was used to control the pain. Lab investigation as in Table 1 revealed a drop in hemoglobin (Hb) from 14 gm/dl to 9 gm/dl, mild thrombocytopenia, hypomagnesemia, hypophosphatemia, hyponatremia, hypokalemia and hypocalcemia. Coagulation studies and lactic acid were within normal range.

**Table 1:** Lab values WBC: White blood cell; PT: Prothrombin time; ALT: Alanine transaminase; AST: Aspartate transaminase; INR: International normalized ratio.

Lab values	
Hemoglobin	9 gm/dl
WBC	11890/mm^3^
Calcium	8.3 mg/dl
PT	12 sec
ALT	50
AST	110
INR	0.95

**Figure 1:**
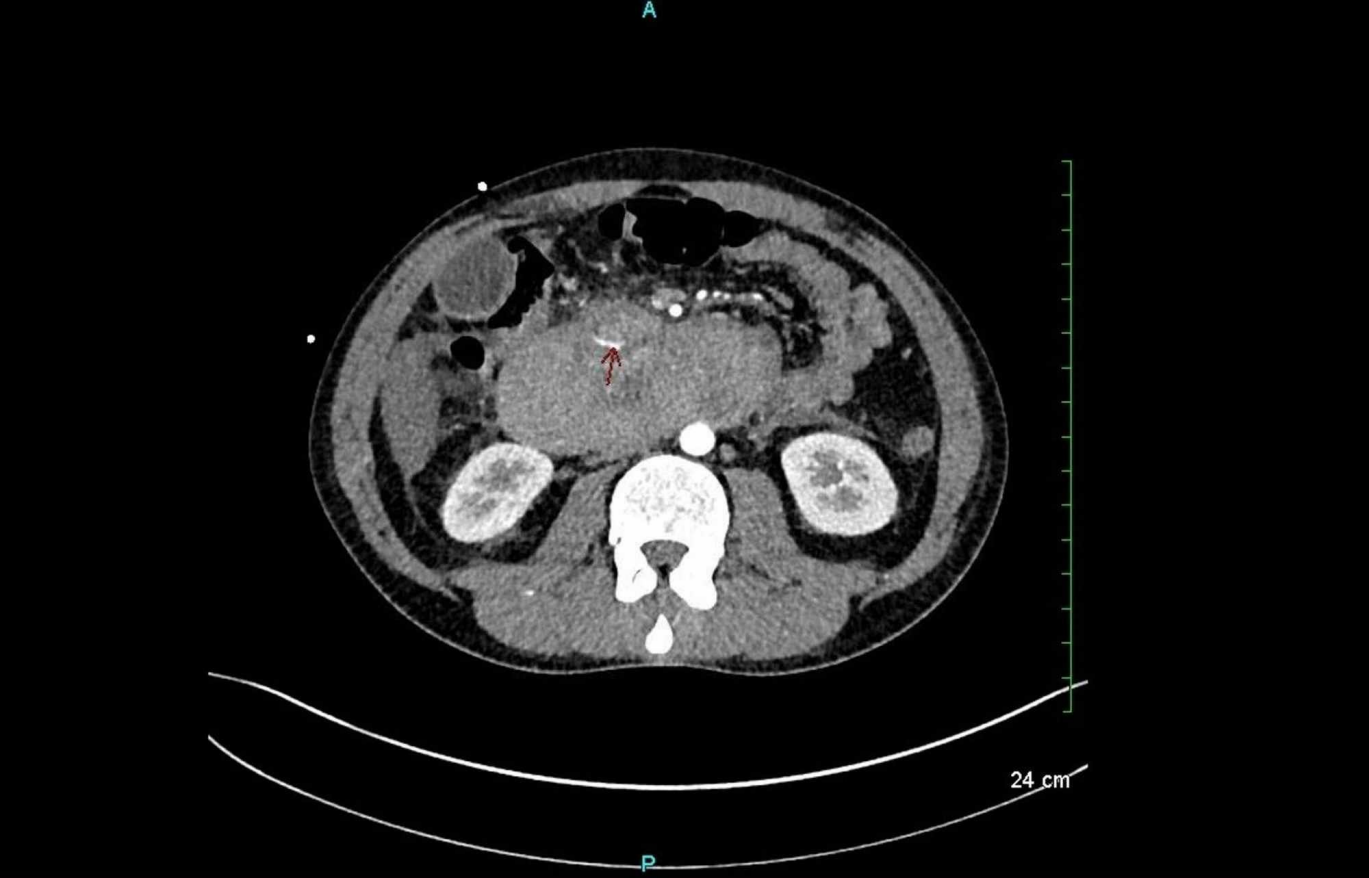
CT scan demonstrating intramural duodenal hematoma.

Chest X-ray and ECG were ordered with assumption of transferring patient for surgery because of presentation with acute abdomen. The patient was evaluated for pancreatitis complications with ultrasonography which showed gallbladder with microlithiasis, sludge and diffuse echogenicity of liver. CT scan showed a 13-cm duodenal hematoma as shown in Figure 1, mild ascites and reactive colitis.

The surgery team was consulted but they refused any intervention due to low hemoglobin and fear of further worsening of hematoma. The patient was managed conservatively with total parenteral nutrition, nasogastric suction, electrolyte correction and pain control. Repeat CT scan a few days later showed regression of hematoma. After 14 days of hospitalization, the patient became asymptomatic and was discharged.

## Discussion

The first case of intramural duodenal hematoma of pancreatic origin was reported in 1938 by Oppenheimer; very few cases have been reported in the past [5]. Pancreatic causes include acute pancreatitis, chronic pancreatitis, ectopic pancreas and pancreatic tumor [6]. This case presented with acute severe abdominal pain while cases in the past had varied presentation with symptoms of gastric outlet obstruction, acute abdominal pain, vomiting and jaundice due to biliary outflow obstruction [7-9]. The hematoma is usually confined inside subserosal layer of duodenum. Pathophysiology remains uncertain although believed to be vascular erosion due to release of proteolytic enzymes from inflamed pancreas. Another hypothesis explains hematoma due to vascular erosion due to ectopic pancreas in the wall of duodenum [9]. CT and MRI remain the most sensitive diagnostic modalities for intramural duodenal hematoma - both are very useful for diagnosis as well as follow-up. Follow-up imaging should be done within two weeks [6]. Management includes conservative measures like fluid resuscitation, pain control and imaging follow-up as performed in this case, because the patient was high risk for the surgery. Arterial embolization is an effective management modality for stable patients [10]. Surgical intervention like laparotomy is reserved for unstable patients. It is a rare complication of pancreatitis which requires further research to establish pathophysiology and prognosis in such cases.

## Conclusions

Intramural duodenal hematoma is a rare condition in itself which is traumatic in origin. Spontaneous intramural duodenal hematoma as a complication of pancreatitis is an even more rare occurrence, and only few cases were found to be reported in the database. Familiarity of this condition is important to establish timely diagnosis and ruling out other complications of pancreatitis.
